# Indigenous Case of Disseminated Histoplasmosis, Taiwan

**DOI:** 10.3201/eid1301.060857

**Published:** 2007-01

**Authors:** Chung-Hsu Lai, Chun-Kai Huang, Chuen Chin, Ya-Ting Yang, Hsiu-Fang Lin, Hsi-Hsun Lin

**Affiliations:** * E-Da Hospital and I-Shou University, Kaohsiung County, Taiwan, Republic of China; † Taichung Hospital of Department of Health, Taiwan, Republic of China

**Keywords:** *Histoplasma capsulatum*, histoplasmosis, rheumatoid arthritis, methotrexate, opportunist infection, Taiwan, dispatch

## Abstract

We report the first indigenous case of disseminated histoplasmosis in Taiwan diagnosed by histopathology of bone marrow, microbiologic morphology, and PCR assay of the isolated fungus. This case suggests that histoplasmosis should be 1 of the differential diagnoses of opportunistic infections in immunocompromised patients in Taiwan.

*Histoplasma capsulatum*, a dimorphic fungus that causes human disease, is endemic in North and Central America, particularly in the region of the Ohio and Mississippi River valleys. Humans are infected by inhalation of the mycelial fragments and microconidia of the organism. After the emergence of HIV infection, histoplasmosis has become 1 of many troublesome opportunistic infections among patients with AIDS. Patients receiving immunosuppressive agents are also predisposed to *H. capsulatum* infection (*1*). Although the organism is found worldwide, cases of histoplasmosis are rarely encountered in Taiwan; only a few, imported, cases have been reported in this decade (*2**–**7*). We report the first indigenous case of disseminated histoplasmosis in Taiwan.

## The Case

In November 2005, a 78-year-old man with underlying rheumatoid arthritis was sent to the emergency department with generalized weakness and poor appetite of several weeks’ duration. He had received oral therapy with prednisolone (5 mg twice per day), hydroxychloroquine (200 mg twice per day), sulfasalazine (1,000 mg twice per day), and methotrexate (MTX) (15 mg per week) for 4 months. The patient’s body temperature was 38.5°C, blood pressure was 129/80 mm Hg, pulse rate was 76 beats/min, and respiratory rate was 20 breaths/min. Physical examination disclosed mild icteric sclera and multiple ecchymosis on the extremities.

A complete blood cell count showed a leukocyte count of 6,110/μL (4% bands, 77% segmented neutrophils, 7% lymphocytes, 6% normoblasts, and 3% myelocytes), hemoglobin level of 11.5 g/dL, and platelet count of 3,000/μL. Biochemical testing showed total bilirubin level of 3.89 mg/dL (normal 0–1.3 mg/dL) and alkaline phosphatase level of 480 U/L (60–220 U/L). Renal function, liver enzymes, and electrolyte levels were all within normal limits. The rheumatoid factor level was 76.3 IU/mL (normal <15 IU/mL). The erythrocyte sedimentation rate and C-reactive protein level were 30 mm/hour (0–20 mm/hour) and 100 mg/L (0–5 mg/L), respectively. Test results for HIV and antinuclear antibody were negative. A chest radiograph showed bilateral interstitial micronodules and a fibrocalcified pattern. Abdominal ultrasonography indicated splenomegaly. The patient was admitted with the tentative diagnosis of MTX-induced thrombocytopenia.

**Figure 1:**
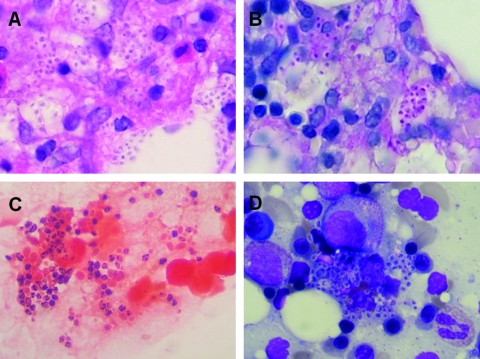
Bone marrow examination of patient A bone marrow biopsy specimen showing numerous oval-shaped intracellular and extracellular microorganisms (A and B). A bone marrow aspiration smear showed numerous intracellular yeastlike microorganisms (C and D). A) hematoxylin and eosin stain, 1,000×; B), periodic acid–Schiff stain, 1,000×; C), Gram stain, 1,000×; and D), Wright stain, 1,000×.

**Figure 2:**
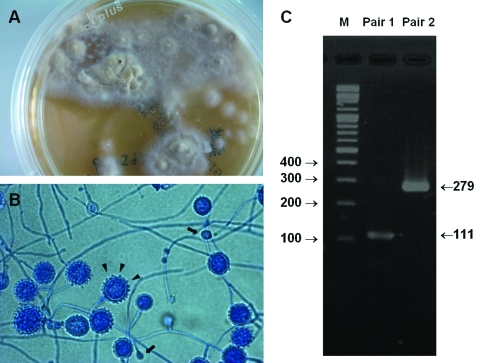
A) Colony of the mold from the patient is white-brown with a cottony appearance on Sabouraud dextrose agar. B) Lactophenol Cotton Blue Stain (Hardy Diagnostics, Santa Maria, CA, USA) of the isolated mold showing thick-walled and tuberculate macroconidia (arrowheads) and microconidia (arrows). C) PCR assay for identification of *Histoplasma capsulatum* based on the nucleotide sequence of the M antigen. PCR products included 111-bp and 279-bp fragments amplified with primers Msp1F-Msp1R (pair 1) and Msp2F-Msp2R (pair 2), respectively, which confirmed the identification of *H. capsulatum* (*8*). M, molecular mass marker.

Bone marrow aspiration (Figure 1) and biopsy were performed because of refractory thrombocytopenia and the presence of young blood cells on the peripheral blood smears. Intravenous amphotericin B (0.7 mg/kg/day) was administered because disseminated histoplasmosis was highly suspected because of the bone marrow findings. Four weeks later, the fungal culture of the bone marrow showed growth of mold (Figure 2A and B). The microorganism was subsequently identified as *H. capsulatum* by PCR assay (Figure 2C) (*8*). No *H. capsulatum* was cultured from the patient’s peripheral blood and sputum.

The patient’s general condition improved after administration of a total dose of 1 g of intravenous amphotericin B, and the patient was discharged and treated with oral itraconazole, 200 mg once a day. Two weeks later, itraconazole therapy was suspended because impaired liver function was found. The patient was closely monitored for 3 months, and no clinical evidence of histoplasmosis relapse was noted.

## Conclusions

A variety of laboratory tests for diagnosis of histoplasmosis, including fungal culture, histopathology, serologic tests, antigen detection, and molecular methods, have different sensitivities based on clinical manifestations and host status (*9*). The standard for diagnosis is isolation of *H. capsulatum* from culture. However, it usually requires ≈4 weeks to grow and has a low sensitivity rate (15%) in self-limited histoplasmosis. The knobby appearance of macroconidia and microconidia indicated by Lactophenol Cotton Blue Stain (Hardy Diagnostics, Santa Maria, CA, USA) of the mold form from fungal cultures is characteristic of *H. capsulatum* (Figure 2B) (*10*). In histopathology, histoplasmosis is impressive, with its numerous ovoid-shaped microorganisms in infected tissue (Figure 1). However, without sufficient experience, one could misidentify *Blastomyces dermatidis*, *Candida glabrata*, *Cryptococcus neoformans*, *Penicillium marneffei*, *Leishmania* spp., *Pneumocystis jirovecii*, or *Toxoplasma gondii* as *H. capsulatum* (*11*). Despite a characteristic form, specific DNA probing is usually applied for faster definitive identification (*12*).

In areas where histoplasmosis is not endemic, including Taiwan, serologic tests, antigen detection reagents, and specific DNA probes for diagnosis of histoplasmosis are not universally available. Among serologic tests, immunodiffusion and complement fixation for anti–*Histoplasma* antibody detection are widely applied; sensitivity rates are 95%–100% and 82%–90% for pulmonary and disseminated histoplasmosis, respectively. Some limitations occur, including the need for 2 to 6 weeks for antibody production after infection, impaired production of antibodies in immunosuppressed patients, and presence of cross-reaction mainly due to paracoccidioidomycosis, blastomycosis, and aspergillosis (*9*). Antigen detection in serum and urine is the most useful method of diagnosing histoplasmosis because it provides early diagnosis before culture and antibody production, monitors response of therapy, and detects relapse. The sensitivity rates of antigen detected are 25%–75% in pulmonary histoplasmosis and 82%–95% in disseminated histoplasmosis. Cross-reactions may occur in cases of paracoccidioidomycosis, blastomycosis, African histoplasmosis, and *P. marneffei* (*9*).

Although the environment in Taiwan is suitable for *H. capsulatum* to grow, histoplasmosis has been rarely encountered. In a survey of histoplasmin skin tests conducted in Taiwan in the 1950s, only 7 (0.19%) of 3,589 schoolchildren tested positive, and the author concluded histoplasmosis probably does not exist in Taiwan or is very rare (*13*). The first possible case of histoplasmosis in Taiwan was reported in 1977; however, the diagnosis was doubtful because it was based only on histopathologic findings in a cervical lymph node biopsy specimen, without definitive fungal culture or molecular identification (*2*). No further cases of histoplasmosis were reported in Taiwan until this decade, when 6 cases were reported (*3**–**7*). The clinical characteristics of these cases are summarized in the Appendix Table. All cases include a history of travel or residence outside Taiwan, where the patients might have acquired the infection; furthermore, most of the patients had underlying HIV infections. Diagnoses were mostly based only on histopathologic or morphologic findings in fungal cultures because the serologic tests, antigen detection reagents, and commercial DNA probes for diagnosis of histoplasmosis were not available in Taiwan. In contrast to these cases, our patient had never traveled outside Taiwan and did not have an HIV infection. To confirm the identification of *H. capsulatum* on the basis of histopathologic and fungal form findings (Figures 1, 2A, and 2B), we applied a specific PCR assay as previously described (*8*), and the results were definitive (Figure 2C). The fibrocalcific nodules found on the chest radiograph imply that either 1) the patient might have had histoplasmosis for years and it became disseminated because of immunosuppressive therapy for rheumatoid arthritis or 2) the patient was reinfected with the calcified lesions that resulted from prior histoplasmosis. The results indicate that this is the first definitive indigenous case of disseminated histoplasmosis in Taiwan. Nonetheless, we were unable to monitor our patient’s response to treatment by antigen tests, as is recommended (*9**,**14*), because they are not available in Taiwan.

The rarity of diagnosed histoplasmosis cases in Taiwan could be explained in several ways. First, the diagnostic rate of histoplasmosis might be markedly decreased because of the lack of serologic and antigen testing kits and reagents, which are useful for diagnosis of self-limited and nondisseminated histoplasmosis. Second, pulmonary histoplasmosis might be misdiagnosed as tuberculosis, which is prevalent in Taiwan. Third, physicians are unfamiliar with histoplasmosis and may consider that histoplasmosis is absent in Taiwan. With increasing immunocompromised hosts resulting from immunosuppressive therapy and HIV infections, as well as improved diagnostic tests, histoplasmosis might be an emergent infectious disease in Taiwan in the future.

In summary, although an indigenous case of histoplasmosis had never been encountered, it should be 1 of the differential diagnoses of opportunistic infections in immunocompromised patients in Taiwan. The true prevalence of histoplasmosis in non-disease–endemic regions might be underestimated because of the paucity of diagnostic tools and familiarity with histoplasmosis.

## Supplementary Material

Appendix Table Clinical characteristics of cases of histoplasmosis reported in Taiwan*
