# Supplementary material for: Indigenous Case of Disseminated Histoplasmosis, Taiwan
Source: Emerg Infect Dis. 2007 Jan;13(1):127–9. doi: 10.3201/eid1301.060857 (PMC2725836; doi:10.3201/eid1301.060857)
Supplement: Appendix Table — Clinical characteristics of cases of histoplasmosis reported in Taiwan* [file 06-0857_appT-s1.pdf]

Lai C-H, Huang C-K, Chin C, Yang Y-T, Lin H-F, Lin H-H. Indigenous case of disseminated histoplasmosis, Taiwan. Emerg Infect Dis. 2007 Jan.

Appendix Table. Clinical characteristics of cases of histoplasmosis reported in Taiwan*

Ref	Age (y)/sex	Year of diagnosis	Manifestation	Signs and symptoms	Chest radiograph	Laboratory examination	Underlying disease	Diagnostic methods	Travel history	Treatment	Outcome
2	32/M	1977	Disseminated histoplasmosis	Cough, backache, neck LAP, anorexia, bodyweight loss	Enlarged hilar lymph nodes	Leukocytosis	None†	Lymph node biopsy only	None	Anti-TB drugs	Died
3	77/M	1994	Laryngeal histoplasmosis	Sore throat, hoarseness	NA	Normal	Old pulmonary TB, adrenal insufficiency	Laryngeal biopsy and fungal culture	Europe, Indonesia, People's Republic of China, Saudi Arabia,	Ketoconazole	Survived
3	37/M	1996	Disseminated histoplasmosis (skin, blood, bone marrow)	Fever and multiple papular skin rash	Normal	Pancytopenia	AIDS (CD4 count: NA), old pulmonary TB, cerebral toxoplasmosis	Skin, bone marrow, liver biopsy and fungal culture	Myanmar	Fluconazole	Died
4	27/M	1997	Gastrointestinal histoplasmosis	Fever, abdominal pain, watery diarrhea, bodyweight loss	Normal	Leukopenic, hypoalbuminemia	AIDS (CD4 count: 200/μL)	Colon tumor biopsy and fungal culture	Thailand, Malaysia, Singapore,	AmB	Survived
5	46/M	1999	Disseminated histoplasmosis (skin, blood, pleural effusion)	Fever, skin ulcers, prolonged diarrhea, jaundice, hepatosplenomegaly	Bilateral pleural effusion	Leukocytosis, anemia, thrombocytopenia, hypercalcemia	Nontyphoid salmonellosis	Skin biopsy and skin, blood, and pleural fungal culture; AccuProbe for pathogen identification	Indonesia	AmB	Died
6	30/M	2000	Disseminated histoplasmosis	Abdominal pain, generalized LAP, hepatosplenomegaly	Normal	Anemia, thrombocytopenia	AIDS (CD4 count: 76/μL)	Stomach and lymph node biopsy	Unknown, but he was a sailor	AmB	Died
7	55/M	2004	CNS histoplasmosis and disseminated TB	Fever, bodyweight loss, and poor appetite, hepatosplenomegaly	Bilateral numerous nodules	Pancytopenia	AIDS (CD4 count: 2/μL)	CSF fungal culture	Myanmar, People's Republic of China	AmB	Died
PR	78/M	2006	Disseminated histoplasmosis	Fever, general weakness, anorexia, splenomegaly	Interstitial micronodules	Anemia, thrombocytopenia	Rheumatoid arthritis treated with methotrexate and prednisolone	Bone marrow aspiration biopsy and culture; PCR assay for pathogen identification	None	AmB	Survived

*Ref, reference; LAP, lymphadenopathy; TB, tuberculosis; NA, data not available; AmB, amphotericin B; CNS, central nervous system; CSF, cerebrospinal fluid; PR, present report.
†HIV examination was not available in Taiwan until the 1980s.

References

1. Deepe GS Jr. *Histoplasma capsulatum.* In: Mandell GL, Bennett JE, Dolin R, editors. Mandell, Douglas, and Bennett's principles and practice of infectious disease. New York: Elsevier; 2005. p. 3012–26.
2. Lee C-H, Chen L, Huang M-J. Disseminated histoplasmosis. Chang Gung Medical Journal. 1977;1:14–8.

3. Kao TW, Hung CC, Hsueh PR, Lin TY, Chen MY, Luh KT, et al. Microbiologic and histologic diagnosis of histoplasmosis in Taiwan. J Formos Med Assoc. 1997;96:374–8.
4. Hung CC, Wong JM, Hsueh PR, Hsieh SM, Chen MY. Intestinal obstruction and peritonitis resulting from gastrointestinal histoplasmosis in an AIDS patient. J Formos Med Assoc. 1998;97:577–80.
5. Liu JW, Huang TC, Lu YC, Liu HT, Li CC, Wu JJ, et al. Acute disseminated histoplasmosis complicated with hypercalcaemia. J Infect. 1999;39:88–90.
6. Lian W-B, Lee Y-T, Lee C-M. Disseminated histoplasmosis in AIDS: a case report. Journal of Internal Medicine of Taiwan. 2000;11:132–8.
7. Hung MN, Sun HY, Hsueh PR, Hung CC, Chang SC. Meningitis due to *Histoplasma capsulatum* and *Mycobacterium tuberculosis* in a returned traveler with acquired immunodeficiency syndrome. J Formos Med Assoc. 2005;104:860–3.
8. Guedes HL, Guimaraes AJ, Muniz MM, Pizzini CV, Hamilton AJ, Peralta JM, et al. PCR assay for identification of *histoplasma capsulatum* based on the nucleotide sequence of the M antigen. J Clin Microbiol. 2003;41:535–9.
9. Joseph Wheat L. Current diagnosis of histoplasmosis. Trends Microbiol. 2003;11:488–94.
10. *Histoplasma capsulatum.* In: Larone DH. Medically important fungi: a guide to identification. 4th ed. Washington: American Society for Microbiology; 2002. p. 150–1.
11. Walsh TJ, Larone DH, Schell WA, Mitchell TG. Histoplasma, blastomyces, coccidioides, and other dimorphic fungi causing systemic mycoses. In: Murray PR. Manual of clinical microbiology. 8th ed. Washington: American Society for Microbiology; 2003. p. 1781–97.
12. Stockman L, Clark KA, Hunt JM, Roberts GD. Evaluation of commercially available acridinium ester-labeled chemiluminescent DNA probes for culture identification of *Blastomyces dermatitidis, Coccidioides immitis, Cryptococcus neoformans,* and *Histoplasma capsulatum.* J Clin Microbiol. 1993;31:845–50.
13. Hsing CT. Histoplasmin and coccidioidin sensitivity among students in Taiwan. J Formos Med Assoc. 1953;4:549–54.
14. Wheat J, Sarosi G, McKinsey D, Hamill R, Bradsher R, Johnson P, et al. Practice guidelines for the management of patients with histoplasmosis. Infectious Diseases Society of America. Clin Infect Dis. 2000;30:688–95.
